# Microbial communities and symbionts in the hard tick *Haemaphysalis longicornis* (Acari: Ixodidae) from north China

**DOI:** 10.1186/1756-3305-6-310

**Published:** 2013-10-28

**Authors:** Li-Meng Liu, Jian-Nan Liu, Zhao Liu, Zhi-Jun Yu, Shi-Qi Xu, Xiao-Hong Yang, Tuo Li, Si-Si Li, Li-Da Guo, Jing-Ze Liu

**Affiliations:** 1 Key Laboratory of Animal Physiology, Biochemistry and Molecular Biology of Hebei Province, College of Life Sciences, Hebei Normal University, Nanerhuan Eastern Road, No. 20, Shijiazhuang, Hebei, P. R. China; 2 Department of Environment and Chemical Engineering, Hebei College of Industry and Technology, Hongqi Road, No. 626, Shijiazhuang, Hebei, P. R. China; 3 College of Life Sciences, Hengshui University, Heping Western Road, No. 1088, Hengshui, Hebei, P. R. China

**Keywords:** *Haemaphysalis longicornis*, Microbial communities, *Coxiella*-like symbiont, *Arsenophonus*-like symbiont, *Rickettsia*-like symbiont

## Abstract

**Background:**

Close relationships between ticks and microbial communities are important for tick fitness and pathogen colonization and transmission. *Haemaphysalis longicornis*, distributed widely in China, can carry and transmit various pathogens and pose serious damages to public health and economics. However, little is known about the broader array of microbial communities and symbionts in *H. longicornis* under natural conditions. In the present study, we investigated the composition of bacterial communities associated with *H. longicornis* and evaluated the putative symbionts*.*

**Methods:**

The eubacterial 16S rRNA gene clone libraries of *H. longicornis* were constructed and analyzed by restriction fragment length polymorphism (RFLP) and DNA sequencing. In addition, diagnostic PCR was performed to assess the prevalence, vertical transmission and infection sites of the symbionts in *H. longicornis*.

**Results:**

Vertically-transmitted symbionts, potential pathogens and allochthonous nonpathogenic bacteria were identified from the field-collected *H. longicornis*. Three types of symbionts (*Coxiella*-like, *Arsenophonus*-like and *Rickettsia*-like symbionts) were identified in a single host simultaneously. A series of analyses revealed the vertical transmission, prevalence, and infection sites of these symbionts. However, only *Coxiella*-like bacteria were transmitted stably in the laboratory-reared ticks. In addition, we identified a novel *Coxiella*-like agent with 95.31% sequence similarity to the taxon described previously.

**Conclusions:**

The present study demonstrated that natural *H. longicornis* harboured a diverse array of microbial communities. Three types of symbionts were identified in a single host simultaneously. Moreover, high prevalence, vertical transmission and the infection sites supported an obligate symbiotic association between *Coxiella* symbiont and its host. The role of *Coxiella* symbiont in the host fitness and the interaction among microbial communities remained to be elucidated. Our investigation of microbial communities in the ticks revealed the complexity of ecological interactions between host and microbe and provided insight for the biological control of ticks.

## Background

As obligate blood-feeding arthropods, ticks feed on terrestrial vertebrates and are one of the most important disease vectors worldwide. Ticks are involved in carrying and transmitting a diverse group of pathogenic bacteria, which exert severely negative impacts on human health, livestock production as well as wildlife [1-3]. Hard ticks (Ixodidae) usually have three life stages (larva, nymph, adult) in their lifespan and feed on distinct host species at different developmental stages. Thus, they are prone to acquiring various pathogens and spreading among vertebrate species during the whole life cycle [4].

In addition to pathogens, vertically-transmitted symbionts and foreign nonpathogenic bacteria also colonize the ticks [5-7]. Symbionts are closely associated with the host fitness [8,9] and transmission and virulence of pathogens [10,11]. To date, it is well-known that ticks harbour various symbionts, including *Coxiella*[12], *Francisella*[13], *Wolbachia*[14], *Rickettsia*[15], *Arsenophonus*[16], *Candidatus* Midichloria mitochondrii [17]. Moreover, some of them have crucial effects on the biological characteristics of ticks. For example, the obligate *Coxiella*-like symbiont manipulates the reproduction of the host *Amblyomma americanum*[18-20]. *Ca.* Midichloria mitochondrii, the symbiont of *Ixodes ricinus*, has the unique ability to enter and destroy mitochondria within ovarian cells of host [21,22], which is coupled with the process of engorgement and molt [23,24]. In addition, colonization of *Dermacentor variabilis* by *R. peacockii* can prevent secondary infection with other *Rickettsia* bacteria, including pathogenic species [25]*.* Clay [5] found that the infection frequency of *Arsenophonus* was negatively correlated to *Rickettsia* in *A. americanum*, indicating that infection with symbionts may affect the microbial community structure and their interaction.

Ticks could also obtain allochthonous nonpathogenic bacteria by sucking host blood and contact with the natural environment [26-28]. Although allochthonous bacteria are not directly related to the pathogenicity of hosts, they can reflect the habitats of hosts and even have the potential to impact on the density and transmission of host-borne agents [29]. Overall, diverse microbial communities can exert great impact on the fitness of ticks and the colonization and transmission of tick-borne pathogens.

*Haemaphysalis longicornis* (Acari: Ixodidae), a three-host tick, extensively distributed in China [30], Korea, Japan, New Zealand, and Australia [31,32], has inflicted serious damage on public health and economics. It is reported that this tick species transmitted a variety of pathogens [33,34], including *Babesia microti*[35], *Ehrlichia chaffeensis*[36], *Anaplasma bovis*[37], *A. phagocytophilum*[38], *C. burnetii*[39], Spotted fever group rickettsiae [40], and *Borrelia burgdorferi*[41]. With regard to the human health and economic significance, pathogens carried by *H. longicornis* have been well investigated in China [42-47]. However, little is known about the broader array of the microbial community in *H. longicornis* under natural conditions.

In the present study, we investigated the composition of bacterial communities associated with *H. longicornis* using PCR-RFLP and DNA sequencing approaches, and evaluated the putative symbionts*.*

## Methods

### Sample collection and storage

*H. longicornis* samples were collected in Xiaowutai National Natural Reserve Area in China by flag dragging. A total of 500 ticks were collected and stored at -80°C. We also collected 1,000 ticks, which were reared on rabbits as described by Liu *et al*. [48]. Colonies of these ticks were reared on the ears of rabbits. Rabbits were maintained in a room with 50-55% relative humidity (RH) at 25–27°C and exposed to daylight. After detachment, ticks were collected and incubated in cotton-plugged glass tubes filled with folded filter paper in an incubator with 75 ± 5% RH and 6/18 h of L/D cycle at 26 ± 1°C. The protocol of all animal experiments was approved by the Institutional Animal Care and Use Committee of Hebei Normal University.

### DNA extraction

The genomic DNA for constructing eubacterial 16S rRNA gene libraries were extracted from a group of adults (10 females and 10 males). To assess the prevalence, vertical transmission and infection sites, DNA was isolated from individual field-collected adults, and pooled ticks at different developmental stages (500 eggs, 200 larvae and 50 nymphs) and different tissues (ovaries, salivary glands, Malpighian tubules and midguts), respectively. Before DNA extraction, the whole tick samples were sterilized as described previously [5], and dissected tissues were washed in sterile phosphate-buffered saline (PBS) (137 mM NaCl, 2.7 mM KCl, 4.3 mM Na_2_HPO_4_ · 7H_2_O, 1.4 mM KH_2_PO_4_, pH 7.4) for three times. Subsequently, all samples were snap-frozen in liquid nitrogen and purified using the DNeasy Tissue Kit (Qiagen, Germany) according to the manufacturer’s instructions.

### Eubacterial 16S rRNA gene library construction, RFLP analysis and sequencing

The eubacterial 16S rRNA gene clone library was constructed by amplifying about 1500 bp fragment of 16S rRNA gene using eubacterial universal primers 27 F/1492R [49]. Purified PCR products were cloned into pEASY-T1 vector (TransGen, China) and transformed into *Escherichia coli* TOP10 competent Cell (TransGen, China). The positive clones were subjected to restriction fragment length polymorphism (RFLP) analyses using both HaeIII and RsaI restriction endonucleases. Sequencing of the positive clones with different restriction fragment patterns were performed by Sangon Biotech Company (China).

### Phylogenetic analyses

The 16S rRNA gene sequences of the most closely related species were retrieved from GenBank and then aligned. The similarity analyses were performed using the CLUSTAL_W program [50]. The phylogenetic tree was constructed using neighbour-joining method implemented in MEGA4.0 [51]. The stability of the tree clustering was evaluated by means of a bootstrap analysis of 1000 datasets [52].

### Diagnostic PCR

To assess their prevalence, vertical transmission and infection site of symbionts, diagnostic PCR assay was performed with four sets of specific primers (Table 1). The PCR reaction contained 20 mM Tris–HCl (pH 8.4), 50 mM KCl, 1.5 mM MgCl_2_, 200 μM each dNTP, 2.5 U Platinum *Taq* DNA Polymerase (Invitrogen, America), and 0.5 mM each primer. PCR cycling conditions were as follows: 1 cycle of 94°C for 2 min; 30 cycles of 94°C for 30 s, 55°C for 30 s, and 72°C for 15 s, and 72°C for 10 min.

**Table 1:** Oligonucleotide primers used for PCR amplification and sequencing

**Primer name**	**Genera or Species**	**Target gene**	**Nucleotide sequence (5′-3′)**	**Annealing temperature (°C)**	**Approx product size (bp)**	**Reference**
CLS F	*Coxiella*	16S rRNA	CACGTAGGAATCTACCTTGTAG	55	90	55
CLS R			CGTTTTGTTCCGAAGAAATTAT			
ALS 82 F	*Arsenophonu*	16S rRNA	AGGGAGCTTGCTTCCTGGCCGG	59	130	55
ALS 198R			CGAAGGTGTGAGGCCTAATGG			
*Rickettsia* 354 F	*Rickettsia*	16S rRNA	CAGCAATACCGAGTGAGTGATGAAG	56	350	69
*Rickettsia* 647R			AGCGTCAGTTGTAGCCCAGATG			
NCLS F	NCLS	16S rRNA	TCCCTGGCGGCGAGTGG	55	110	This study
NCLS R			CGTATTAGAGATTAGAGAAACC			
*Rr*190.70p	*Rickettsia*	*rompA*	ATGGCGAATATTTCTCCAAAA	48	540	57
*Rr*190.602n			GTTCCGTTAATGGCAGCATCT			

## Results

### Diversity of bacterial communities from field-collected *H. longicornis*

Our results revealed diverse microbial communities associated with field-collected *H. longicornis* (Table 2). In the 16S rRNA gene clone libraries, the most abundant sequences (belonged to *Coxiella* and designated as CLS-Hl) in both males and females share 99.5% similarity to the symbiont of *H. longicornis* (GenBank: AB001519) [53]*.* In females, the second abundant sequence (designated as NCLS-Hl) belonged to *Coxiella*, sharing 95.3% similarity to *Coxiella*-like symbionts of *Rhipicephalus sanguine* (GenBank: D84559)*.* Following NCLS-Hl, sequences belonged to *Rickettsia* (designated as RLS-Hl) and *E. coli. Rickettsia* bacteria shared the highest sequence similarity (99.8%) with symbiotic *Rickettsia* of *D. varibilis* (GenBank: U55820) [54]. In males, the second abundant microbes (designated as ALS-Hl) belonged to *Arsenophonus*, sharing over 99.5% and 97.9% similarity to symbionts of *D. silvarum* (GenBank: JN866582) [55] and *D. variabilis* (GenBank: AY265342) [56], respectively*.* Following ALS-Hl, there were RLS-Hl and NCLS-Hl. Interestingly, the top four abundant bacteria in females were consistent with those in males.

**Table 2:** **Eubacterial 16S rRNA gene clone libraries from females and males of *Haemaphysalis longicornis***

**Classification**	**Closest taxon (GenBank No.)**	**Identity %**	**Sequences**^**# **^(F/M)*	**Accession No. (GenBank)**
**Proteobacteria**				
α-proteobacteria	*Rhizobium* spp. (EF363715)	99%	4/0	JN866565
	*Rickettsia* spp. (CP003319)^§^	99%	9/12	JN866571
	*Devosia psychrophila* (GU441678)	98%	0/1	JN866597
	*Asticcacaulis excentricus* (CP002395)	99%	2/0	JN866569
β-proteobacteria	*Janthinobacterium lividum* (EU275366)	99%	0/5	JN866578
γ-proteobacteria	*Escherichia coli* (CP001673)	100%	8/7	JN866566
	*Moraxella osloensis* (AB643592)	99%	4/0	KF421818
	*Pseudomonas* spp*.* (AB334768)	99%	0/7	JN866577
	*Coxiella*-like symbionts of *H. longicornis* (AB001519)^§^	99%	36/19	JN866564
	*Coxiella*-like symbionts of *R. sanguine* (D84559)^§^	95%	21/11	JN866567
	*Arsenophonus*-like symbionts of *Dermacentor silvarum* (JN866582)^§^	99%	5/16	JN866572
**Bacteroidetes**				
Sphingobacteria	*Mucilaginibacter* sp. (GU139695)	98%	1/0	JN866568
	*Sphingomonas asaccharolytica* (AY509241)	98%	0/1	JN866579
**Actinobacteria**				
Actinobacteria	*Williamsia maris* (NR024671)	99%	0/2	JN866598
	*Conexibacter woesei* (CP001854)	98%	0/1	JN866581

^*^Source of the sequences from female ticks (F) or male ticks (M).

^#^Sequences indicate the number of 16S rRNA gene sequences obtained for each 16S rRNA gene clone libraries.

^§^Sequences are related phylogenetically to previously reported tick-associated bacteria.

### Symbionts

**Figure 1:**
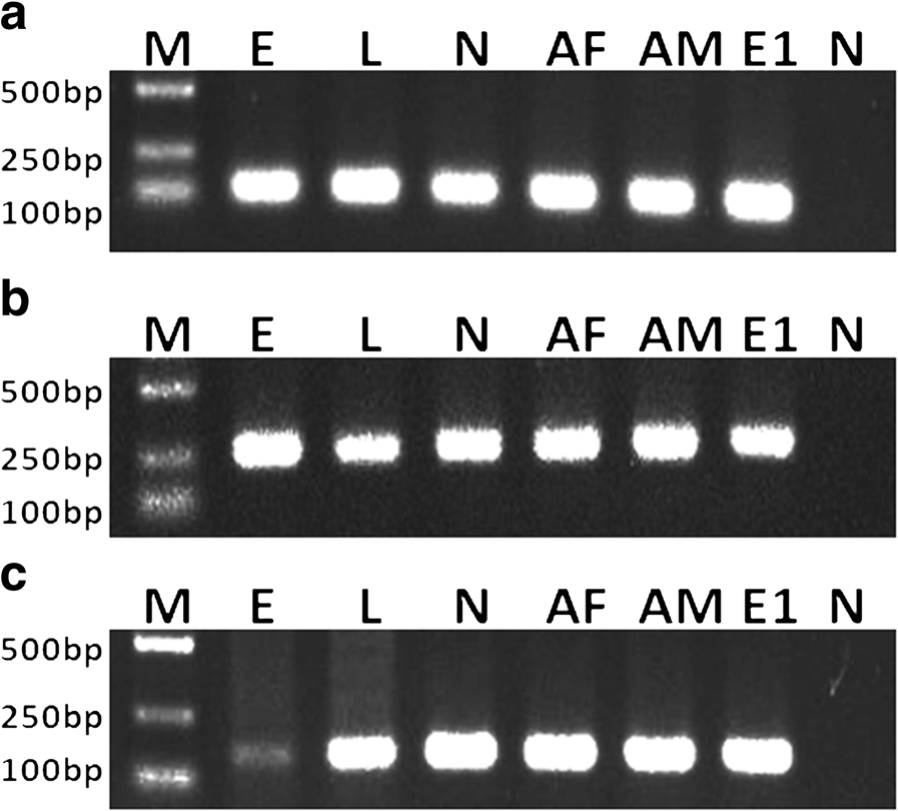
**Detection of vertical transmission of CLS-Hl (a), RLS-Hl (b) and ALS-Hl (c) by diagnostic PCR amplification.** Lanes 1 to 8: M, DNA ladder; E, eggs from field-collected females; L, larvae; N, nymphs; AF: adult females; AM: adult males; E1: eggs from lab-reared females; N, negative control (distilled water).

*OmpA* gene sequence analyses revealed that RLS-Hl belonged to *Ca.* Rickettsia hebeiii, with 99.1% sequence similarity [57]. To confirm vertical transmission of symbionts, eggs and the first generation of laboratory-reared ticks (larvae, nymphs and adults) was tested by diagnostic PCR assay. The putative symbionts were found in all samples detected, with the exception of NCLS-Hl. These results indicated that CLS-Hl, ALS-Hl and RLS-Hl were inherited from progeny to the next generation, while NCLS-Hl was not (Figure 1). To determine the stability of vertical transmission, we investigated the presence of each bacterium in the 7th generation of laboratory-reared ticks (F7). The results showed only CLS-Hl was detected in F7 ticks, while ALS-Hl and RLS-Hl were not, suggesting that CLS-Hl can be maintained stably in laboratory-reared ticks.

In natural field populations, CLS-Hl showed 100% infection rate; ALS-Hl occurred in 33.3% (14/36) females and 83.3% (10/12) males; the prevalence of RLS-Hl was 88.9% (32/36) and 100% (12/12) in females and males, respectively. In the laboratory population, the prevalence of CLS-Hl was 100%.

**Figure 2:**
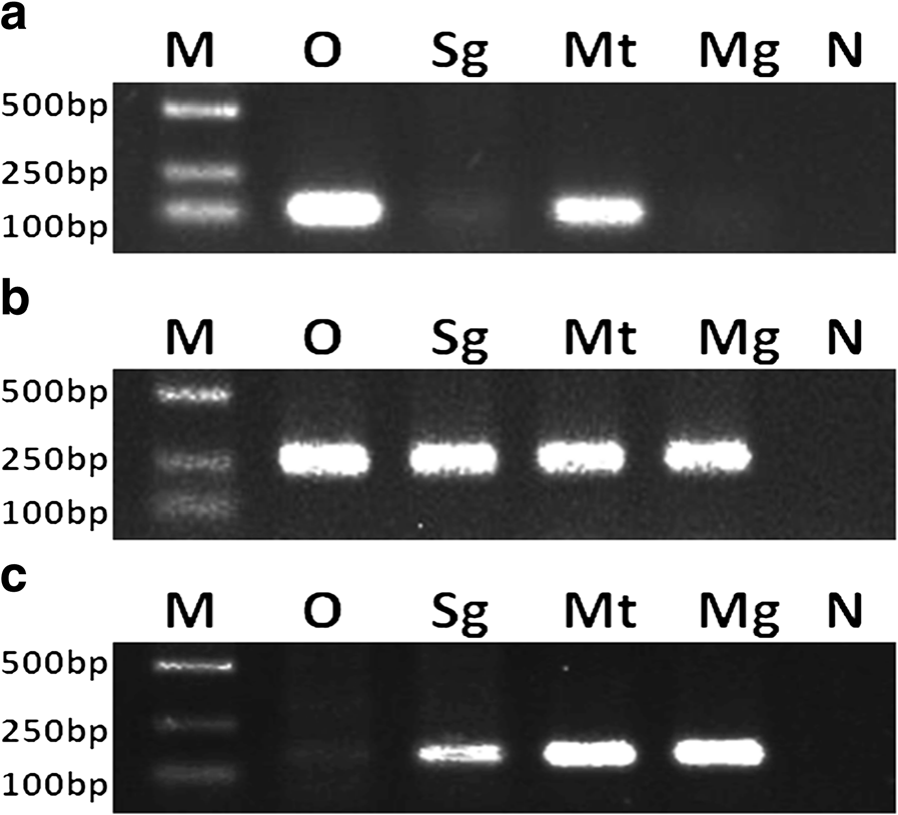
**Detection of infection sites of CLS-Hl (a), RLS-Hl (b) and ALS-Hl (c) by diagnostic PCR amplification.** Lanes 1 to 6: M, DNA ladder; O, ovaries; Mt, Malpighian tubules; Gs, salivary glands; Mg, midguts; N, negative control (distilled water).

Analysis of the infection site showed that three types of symbionts infected all tissues detected (accessory glands, testes, salivary glands, midguts) and there is no significant difference among all tissues in males. In females, CLS-Hl colonized specifically in the ovaries and Malpighian tubules, while ALS-Hl and RLS-Hl infected all tissues tested (ovaries, salivary glands, Malpighian tubules and midguts) (Figure 2).

## Discussion

In this study, we showed that the natural *H. longicornis* harboured a diverse group of bacterial species. Previous studies have shown that some tick species were colonized with complex microbial communities, including *A. americanum*, *I. ricinus*, *I. scapularis* and *Rh. microplus*[5-7,26-28,58,59]. Investigation of microbiota in the ticks revealed the complexity of ecological interactions between host and microbe and provided insight for the biological control of ticks.

### Symbionts

**Figure 3:**
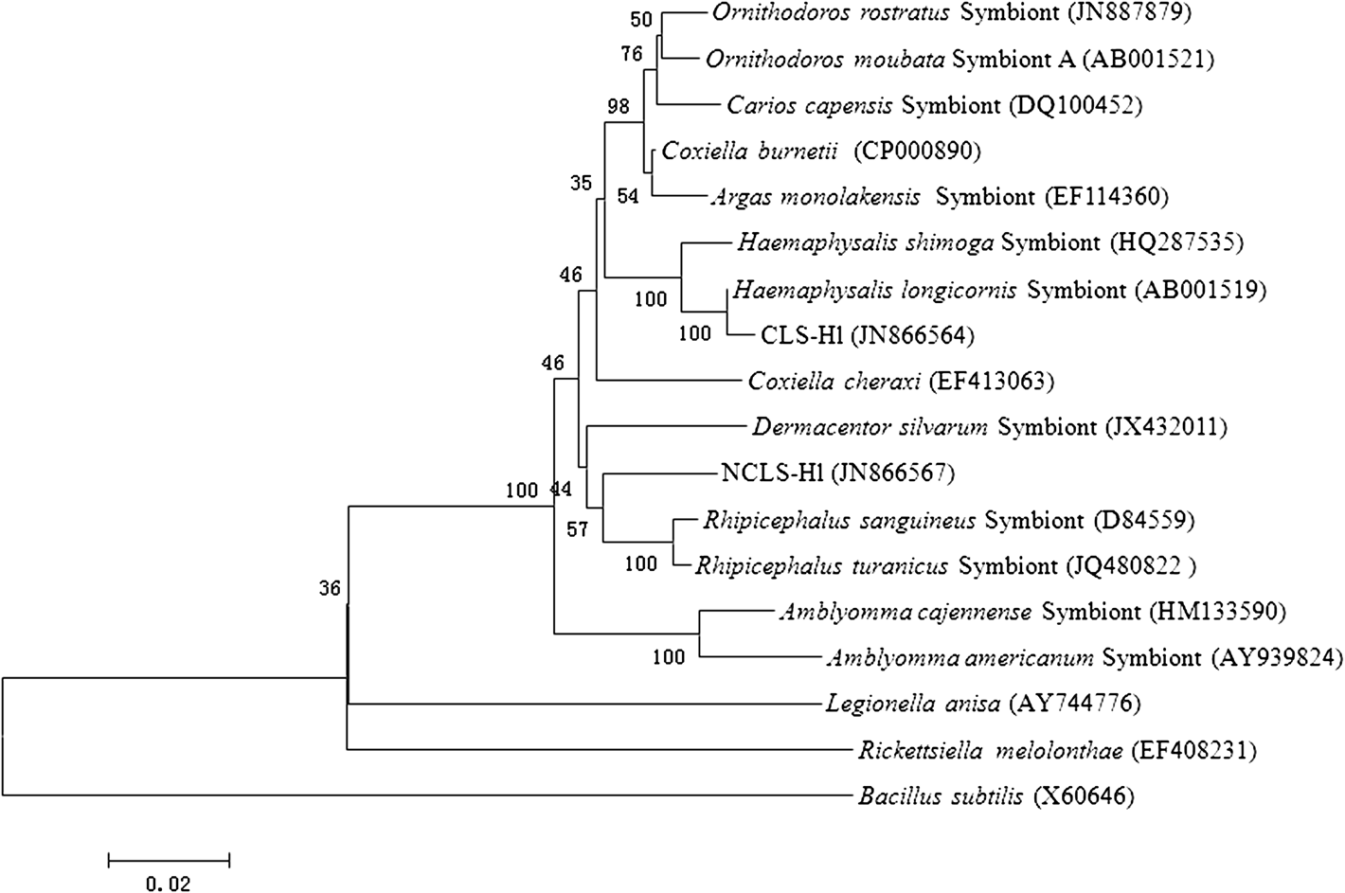
**Phylogenetic tree of two types of *Coxiella*-like bacteria based on 16S rRNA gene sequence similarity.** The tree was rooted with *Bacillus subtilis* (GenBank: X60646) and constructed using neighbour-joining method and clustering nodes were also recovered in maximum likelihood method. Numbers at nodes represent the levels of bootstrap support (%) based on neighbour-joining analysis of 1000 replicated data sets. GenBank accession numbers are given in parentheses. Bar represents 2% sequence divergence.

Interestingly, some sequences were phylogenetically related to previously reported tick-associated bacteria, including (1) two types of *Coxiella*-like bacteria, CLS-Hl and NCLS-Hl, (2) *Arsenophonus*-like symbiont, ALS-Hl, (3) *Rickettsia*-like symbiont, RLS-Hl. It is now widely accepted that the co-infection with multiple symbionts occurs in many arthropod hosts [60]. However, co-infection with symbionts has been demonstrated only in a few tick species, such as *A. americanum*[5], *Ornithodoros moubata*[61], *D. silvarum*[55] and *Rh. sanguineus*[62]. We reported here for the first time that *Coxiella*-like, *Arsenophonus*-like and *Rickettsia*-like symbionts were detected simultaneously in wild *H. longicornis*. Interestingly, we identified two different types of *Coxiella*. CLS-Hl, with 95.1% similarity to the *C. burnetii*, shared 99.5% sequence identify with the symbionts of *H. longicornis* previously reported [39,53]. However, NCLS-Hl, with 95.1% and 95.3% similarity to the *C. burnetii* and symbionts of *Rh. sanguine*, respectively, was regarded as a novel *Coxiella*-like agent and has never been described before. Phylogenetic analyses revealed (Figure 3) that CLS-Hl and NCLS-Hl form an individual clade, although both of them belong to the cluster of hard tick species (Ixodidae). CLS-Hl was clustered with symbionts of *Haemphysalis* (*H. longicornis* and *H. shimoga*), while NCLS-Hl was grouped together with the symbionts of *Rhipicephalus* (*Rh. sanguineus* and *Rh. turanicus*). However, the determination of vertical transmission does not support that NCLS-Hl was a symbiont. Further studies are needed to confirm the pathogenicity of this novel *Coxiella* agent to vertebrate hosts.

In ticks, *Coxiella* bacteria are the most common agent, exhibiting diverse genotypes in different tick species [5,12,63-68]. By comparing the phylogeny between the tick mitochondrial 16S rRNA gene and the *Coxiella* agents carried by ticks, Almeida [12] showed that ticks were phylogenetically associated with their *Coxiella* agents, suggesting that *Coxiella* symbionts and ticks shared a long co-evolution process. In *H. longicornis*, *Coxiella* symbionts from different areas, such as Japan [53] and Korea [39], shared identical genotypes. This wide geographical distribution indicates that *Coxiella* symbionts form ancient symbiotic associations with their host before spreading.

Previous studies suggested that *Coxiella* symbionts in *A. americanum*, with 100% infection, vertical transmission, reduced-genome, regulation of the reproduction of the host, was closely related to the fitness of the ticks. Besides, in *O. rostratus*[12], *D. silvarum*[55] and *Rh. sanguineus*[62], *Coxiella*-like symbionts exhibited nearly perfect prevalence and were presumed as obligate symbionts. In this study, 100% infection rate, perfect vertical transmission, colonization in specialized tissues, CLS-Hl was regarded as obligate and contributed to the fitness of ticks.

*Arsenophonus* is one of the four major inherited symbionts of arthropods with an infection rate of 5% [60]. *Arsenophonus* has been reported to infect *A. americanum*[5], *D. variablilis*[16], *D. andersoni*[16] and *D. silvarum*[55]. Interestingly, the sequence of ALS-Hl was identical to that of *D. silvarum* collected from the same place as the *H. longicornis*, possibly suggesting horizontal transmission of *Arsenphonus*-like symbionts between different genera of ticks. Previous studies also provided evidence of horizontal transmission of symbionts between *D. variabilis* and *D. andersoni*[16]. Therefore, *Arsenophonus* possessed not only intragenus, but also intergenus horizontal transmission.

*Rickettsia*, with both symbiotic and pathogenic lifestyle, inhabits multiple tick species. Pathogenic rickettsiae have been extensively investigated, while only few studies on symbiotic rickettsiae have been reported. To date, *Rickettsia*-like symbionts have been identified in *D. variabilis*[54]*, D. andersoni*[69]*, D. silvarum*[55]*I. scapularis*[15] and *A. americanum*[5]. In this study, we showed molecular evidence for the vertical transmission of the *Rickettsia*-like symbiont (*Ca.* Rickettsia hebeiii) in *H. longicornis*. Moreover, high prevalence in the natural environment suggested interaction between RLS-Hl and their hosts.

However, RLS-Hl and ALS-Hl were not detected in F7 laboratory-reared ticks, suggesting that they cannot be maintained stably from one generation to the progeny in the laboratory. This phenomenon was also observed in *I. ricinus*, whose symbionts IricES1 were lost from female adults to progeny in the laboratory [70]. The transmission efficiency of symbionts decreased significantly in the laboratory, which was partially due to the competition among symbionts and bottlenecks during vertical transmission [71]. Alternatively, transmission reduction may be due to temperature differences between natural and laboratory environments [72].

## Conclusions

A diverse array of microbial communities were identified from field-collected *H. longicornis*. Three types of symbionts were detected in a single host simultaneously. Moreover, analysis on the prevalence, vertical transmission and infection sites supported obligate symbiotic association between *Coxiella* symbionts and its host. The role of *Coxiella* symbionts in the host fitness and the interaction among microbial communities remains to be elucidated. Our investigation of microbial communities in the ticks revealed the complexity of ecological interactions between host and microbe and provided insight for the biological control of ticks.

## Abbreviations

F7: The 7th generation of laboratory-reared ticks; CLS-Hl: *Coxiella*-like symbiont of *H. longicornis*; NCLS-Hl: Novel *Coxiella*-like symbiont of *H. longicornis*; RLS-Hl: *Rickettsia*-like symbiont of *H. longicornis*; ALS-Hl: *Arsenophonus*-like symbiont of *H. longicornis.*

## Competing interests

The authors declare that they have no competing interests.

## Authors’ contributions

Liu L-M and Liu J-Z conceived and designed the study, constructed 16S rRNA gene libraries and performed RFLP analyses, drafted the manuscript, and critically revised the manuscript. Liu J-N carried out RFLP analyses and diagnostic PCR. Liu Z, Yu Z-J, Xv S-Q and Yang X-H, participated in sample collection, study implementation and data collection and helped to revise the manuscript. Li T, Li S-S and Guo L-D participated in sample collection and tick feeding. All authors read and approved the final manuscript.
